# Clinical and demographic characteristics of adolescents, emerging adults, and young adults presenting to an emergency department following a suicide attempt

**DOI:** 10.1002/jcv2.70044

**Published:** 2025-08-30

**Authors:** Nicholas M. Brdar, Natasha K. Matta, Chloe A. Miner, Mubashshir R. Bhuiyan, Irmgard G. Pallas, Anna L. Bickersteth, Hailey G. Prokop, Lindsay A. Bornheimer

**Affiliations:** ^1^ School of Social Work University of Michigan Ann Arbor Michigan USA; ^2^ Department of Psychiatry Michigan Medicine Ann Arbor Michigan USA; ^3^ University of Michigan College of Literature, Science, and the Arts Ann Arbor Michigan USA

**Keywords:** adolescent, emerging adult, risk assessment, suicide, suicide attempt

## Abstract

**Introduction:**

Suicide is a significant public health concern and the second leading cause of death in adolescents and young adults. This study sought to better understand differences in demographic and clinical characteristics of young people who attempted suicide during three phases of this developmental period: late adolescence (14–17 years), emerging adulthood (18–25 years), and young adulthood (25–29 years).

**Methods:**

Demographic and clinical characteristics, including suicide attempt and hospital encounter details, were collected from the Electronic Health Record of 1706 patients ages 14–29 who presented to an academic healthcare system in the Midwestern United States between 2008 and 2023 after a suicide attempt. Differences between age groupings were examined using Chi‐square tests and one‐way ANOVAs.

**Results:**

Overall, patients were an average age of 18.86 years old (SD = 4.19) with a majority identifying as white (75.5%), female (68.3%), cisgender women (63.5%). Significant differences in demographic and clinical characteristics were found across age groups, including: gender, race, sex, attempt method, length of hospital stay, suicide risk assessment, and discharge disposition. Of note, despite endorsing higher levels of suicide risk severity, emerging adults were disproportionately determined to be at a moderate risk level and had shorter lengths of stay in the hospital.

**Discussion:**

Findings expand upon literature that distinguishes emerging adulthood as a distinct developmental period from adolescence and adulthood, in addition to highlighting the complexities of identifying and managing suicide risk during this developmental period. Implications point toward the need to tailor suicide prevention approaches for emerging adults, target implementation efforts to specifically reach this population, and ensure resources are provided in alignment with their developmental needs.

## Key points

**What's known?**

- Suicide is a leading cause of death for youth and young adults, and studies focusing on those who have attempted suicide may provide insight that can help inform future prevention efforts.

**What's new?**

- In the present study, patients in each of the three age groups significantly differed in a number of demographic and clinical characteristics, including gender, race, sex, attempt method, non‐suicidal self‐injurious behavior, interrupted/aborted attempt, history of prior attempt, length of hospital stay, suicide risk assessment, and discharge disposition.
- Findings reinforce and build upon existing literature that establishes emerging adulthood as a distinct developmental period, suggesting a need for more developmentally‐relevant assessment and intervention for this population.

**What's relevant?**

- Study implications emphasize the need for tailored suicide prevention approaches with unique considerations for implementation among transitional aged youth and young adults to ensure resources are provided in alignment with their developmental needs.

## INTRODUCTION

Suicide is a significant public health concern and ranks among the top causes of death in the United States (Centers for Disease Control and Prevention [CDC], 2024a, 2024b). Of particular concern, suicide is the second leading cause of death in adolescents and young adults ages 10–34 (Garnett et al., 2023), accounting for over 14,000 deaths annually (CDC, 2024a, 2024b). Rates for suicide thoughts and behaviors are also concerningly high among young people, with data indicating 13% of young adults ages 18–25 have had serious thoughts about suicide in the past year and (Substance Abuse and Mental Health Services Administration [SAMHSA], 2024) and nearly 10% of high school‐aged adolescents have attempted suicide in the past year (CDC, 2024a, 2024b). Despite the higher prevalence of suicide thoughts and behavior (i.e., attempt) among transition‐aged youth and young adults than in adult populations (MacKinnon & Colman, 2016), young people are less likely to seek professional help for their experiences (Junus et al., 2022; MacKinnon & Colman, 2016). The high rates of suicide ideation, behavior, and death, paired with reduced help‐seeking, necessitate a closer investigation of risk and protective factors associated with suicide attempt during the transition from adolescence into early adulthood.

Existing literature has revealed numerous risk factors for suicide death in youth and young adults, including: depressive symptoms (Busby Grant et al., 2023; Gili et al., 2019), interpersonal losses (Spirito & Esposito‐Smythers, 2006), familial conflict (Gould et al., 2003; Soole et al., 2015), and substance use (Rioux et al., 2021); all of which can be exacerbated by the pressures of life changes including emerging responsibilities and shifts in education, employment, and housing (MacKinnon et al., 2016). This myriad of lifestyle changes and novel stressors (e.g., social, educational, professional) that hallmark the developmental transition to adulthood have been shown to potentially increase one's risk for suicide thoughts and behavior (MacKinnon et al., 2016). In recent decades, there have also been societal shifts such that milestones typically achieved upon reaching adulthood (e.g., marriage, parenthood, entering the workforce, financial independence) are more often being delayed until the mid‐to‐late twenties (Arnett, 2000). In response to these shifts, Arnett (2000) proposed a novel developmental stage, coined “emerging adulthood,” which spans ages 18–25 and is characterized by in‐depth identity exploration, shifts in peer and family relationships, and increased autonomy and responsibility as an individual transitions from adolescence to adulthood (Arnett, 2000).

Literature further indicates that inadequate access to mental health care and resources can lead to adverse mental health outcomes in young adults, especially among emerging adults (Khetarpal et al., 2022). With the prevalence of mental illness being highest among emerging adults ages 18–25 (36.2%) compared to adults ages 26–49 (29.4%) and adults ages 50 and older (13.9%), a smaller proportion of emerging adults seek treatment compared to their older counterparts (SAMHSA, 2024). This period is further complicated by novel barriers to mental healthcare, including difficulty navigating the adult mental health care system, unfamiliarity with adult mental health care providers, lack of support transitioning from school‐based care after graduation from high school, and the need for more independence and responsibility for their health and medical decisions (Khetarpal et al., 2022). Some young adults also fall through the transition gap between pediatric and adult healthcare settings resulting in discontinuity in care, unmet mental health needs, and increased distress, anxiety, and frustration (Appleton et al., 2022).

Despite societal shifts and heightened barriers to care, research has traditionally investigated suicide thoughts and behaviors among adolescents and young adults either together as one population (i.e., adolescents and young adults), or separately as pediatric and adult populations. This may, in turn, limit the ability for risk to be identified and understood among young people transitioning from adolescence through emerging adulthood into adulthood. As such, this present study sought to explore comparisons between three groupings of ages to represent late adolescence (Adol; 14–17 years), emerging adulthood (EA; 18–25 years), and young adulthood (YA; 25–29 years). Specifically, we aimed to evaluate potential differences between clinical and demographic characteristics among those who made a suicide attempt in late adolescence (Adol), EA, and YA. It is hypothesized that demographic and clinical characteristics would significantly differ across age groups. Given the various lifestyle changes, stressors, and high prevalence of mental illness that may increase risk for suicide specifically during EA, we anticipated that the characteristics of EA would differ from both Adol and YA groups, further suggesting the need to consider EA as a distinct developmental period in need of further attention in suicide prevention research and clinical practice.

## METHODS

Data were obtained from Electronic Health Record (EHR) of patients aged 14–29 who presented to an academic healthcare system in the Midwestern United States after making a suicide attempt between 2008 and late 2023. Inclusion criteria of the current study were: (1) being 14–29 years of age at time of encounter and (2) presenting to the hospital within 24 h of making a suicide attempt. Exclusion criteria included: (1) presenting suicidal ideation, intent, and/or planning without engaging in any suicide behavior; (2) presenting for non‐suicidal self‐injurious behavior; or 3) presenting to the hospital more than 24 h after making a suicide attempt. This study was approved by the University of Michigan Institutional Review Board (IRB).

### Sample selection

**FIGURE 1:**
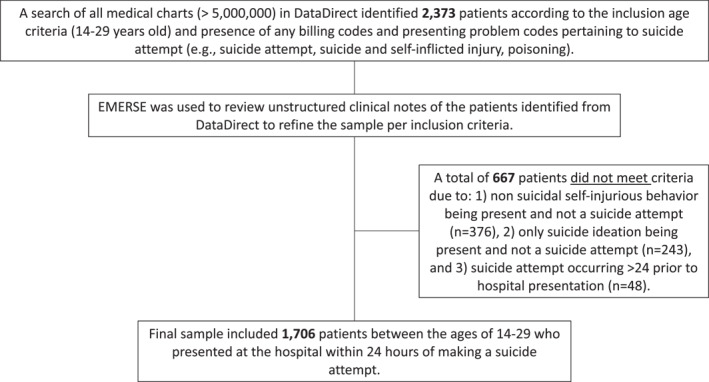
Study flow diagram of sample identification and eligibility determination.

As illustrated in Figure 1, a broad search was conducted as a first step using DataDirect, a cohort selection tool that uses structured clinical data (i.e., diagnoses, encounters, medications, billing codes) to identify cohorts of patients for research investigations from a database of over 5,000,000 medical records. In addition to the age criteria (14–29 years old), all billing codes and presenting problem codes that may have pertained to a suicide attempt were included in the search (e.g., suicide attempt, suicide and self‐inflicted injury, poisoning), yielding 2373 patient medical record numbers (MRNs).

Next, these MRNs were inputted to an Electronic Medical Record Search Engine (EMERSE; Hanauer et al., 2015) which allows for access and review of the unstructured clinical data in the EHR (i.e., provider notes). EMERSE aids in identifying relevant encounter notes by suggesting and highlighting synonyms of search terms to capture potentially relevant encounters or details within provider notes, such as alternate verb tenses or typical misspellings. For example, a search of the term “suicide” resulted in 103 related search terms, including “suicidal,” “sucide,” “end their life,” and “ended their life”. After using EMERSE to review the EHR of potentially eligible patients, a total of 667 individuals were excluded based on the criteria above, most often due to presenting for non‐suicidal self‐injury (*n* = 376, 56.4%). This resulted in a final analytic sample of 1706 patients.

### Data collection

Once eligibility was determined, trained RAs reviewed EHR documentation of each patient using a structured data collection protocol. For those with more than one suicide attempt encounter in the EHR, data collection focused on the patient's most recent attempt given chart documentation grows in comprehensiveness over time. Data were primarily collected from encounter summary notes written by medical doctors and/or social workers in the children's, adult, and psychiatric emergency departments (EDs). For those who were admitted beyond the ED, data were also collected from the history and physical (H&P) and discharge summary notes written by medical doctors. Lastly, data were also collected from previous encounter notes when applicable to ensure documentation of historical data or data not documented in the current encounter notes (e.g., history of attempt and demographic characteristics).

Data regarding demographic characteristics (e.g., age, race, sex, gender), clinical experiences (e.g., mental health symptoms and diagnoses, suicide attempt risk and protective factors, suicide intent), suicide attempt details (e.g., precipitating event, method, post‐attempt injuries and symptoms), and hospital encounter information (e.g., length of stay [LOS], admission status, encounter trajectory throughout hospital including and beyond the ED) were collected. As part of the encounter trajectory, RAs coded a patient's encounter with specificity given this academic health system has multiple EDs (adult, pediatric, and psychiatric), and it is not uncommon for patients to visit multiple EDs in the same encounter (e.g., transfer to the psychiatric ED after medical clearance in the pediatric ED).

Scores documented in the EHR of the Columbia‐Suicide Severity Rating Scale (C‐SSRS; Posner et al., 2011) were also collected. C‐SSRS items pertain to passive ideation (wish to be dead), active ideation (active suicide thoughts), active ideation with a method, some intent to act (without specific plan), and suicide plan. In alignment with an established scoring and analysis guide (Nilsson et al., 2013) responses were coded as current experiences (i.e., current ideation) with binary responses (yes/no) and a numerical score was derived as a sum of items with a range of 0–5. The C‐SSRS has documented good convergent and divergent validity with other suicide ideation and behavior scales, and has been shown to be reliable in prior research with a Cronbach's alpha of 0.93 (Posner et al., 2011). A Chronbach's alpha of 0.86 was found in the current study.

After data were collected, several variables were re‐coded into categorical groupings. Age is of central focus in the current study and therefore the continuous variable was coded to represent 3 groupings: adolescents (Adol; ages 14–17), emerging adults (EA; ages 18–25), and young adults (YA; ages 26–29). Two variables related to medical severity of the suicide attempt were also recoded by a resident physician: injuries/symptoms and medical intervention. Injuries and symptoms resulting from the attempt were categorized into the following by a medical doctor: none, low (e.g., superficial burns, changes in blood pressure or heart rate), moderate (e.g., deep lacerations, cyanosis, oxygen desaturation), and high (e.g., comatose, cardiac arrest). Medical intervention after the attempt was similarly categorized into levels of severity: none, low (e.g., saline drip), moderate (e.g., sutures, narcan administration), and high (e.g., surgery, intubation). Lastly, providers documented a total of 194 unique risk factors for suicide attempt, and these were separated in the risk assessments into the following categories: static (e.g., history of suicide attempt, history of abuse), modifiable (e.g., social isolation, intense anxiety), and imminent (e.g., belief in lethality of plan, access to firearms). These risk factors were examined individually and by grouping.

### Data analysis

Data were analyzed using SPSS 28. Univariate explorations of demographic and clinical characteristics were performed to describe the sample. Differences in demographic and clinical characteristics between age groupings (Adol, EA, and YA) were examined using Chi‐square tests for categorical and one‐way ANOVAs for continuous demographic and clinical characteristic variables. Post‐hoc testing was performed for ANOVAs using Tukey post‐hoc testing (with eta‐squared (*η* ^2^) effect sizes), and Chi‐square analyses using Bonferroni Pairwise Comparison of Proportions (with Cramer's V effect sizes). We anticipated a degree of missingness for some demographic or clinical characteristics that are not required as response fields by providers in the EHR. Still, the vast majority of variables (88%) had less than 2% missing data for individual cases. As for missing data, three variables (i.e., race, suicide intent, suicide risk level determination) had 2%–10% missing data, and one variable (sexuality) had 70% missing data.

## RESULTS

### Full sample demographic and clinical characteristics

Demographic characteristics of the sample are presented in Table 1. Patients were 18.86 years of age (Standard Deviation; SD = 4.19) on average, and the majority identified as non‐Hispanic/Latinx (*n* = 1,567, 93.5%) and white (*n* = 1,261, 75.5%). Most patients were female (*n* = 1,165, 68.3%) and identified as cisgender women (*n* = 1,073, 63.5%), followed by cisgender men (*n* = 494, 23.2%) and transgender or nonbinary (*n* = 123, 7.3%). The vast majority of patients had previously been diagnosed with a mental health condition (*n* = 1,574, 92.3%), most often depression (*n* = 1,271, 75.1%), anxiety (*n* = 866, 50.8%), and ADHD (*n* = 436, 25.5%). Slightly more than half of the patients endorsed a history of suicide attempt (*n* = 898, 55%) prior to the encounter of focus in this study, and of those patients, the majority (*n* = 613, 68.2%) endorsed making 2–5 prior attempts.

**TABLE 1:** Demographic characteristics by age group.

	Full sample (*n* = 1706)		Adolescents (*n* = 873, 51.2%)		Emerging adults (*n* = 656, 38.5%)		Young adults (*n* = 177, 104%)		Significance^a^			
	*n*	%	*n*	%	*n*	%	*n*	%	*F* or *χ* ^2^	*df*	*p*	Post hoc^b^
Age in years (M, SD)	18.86	4.19	15.6	1.1	20.9	2.2	27.6	1.2	4773.70	2	<0.001	a,b,c
Race									7.066	2	0.006	
White	1261	73.9	666	82.8	465	75.6	130	76.9				a
Black	226	13.2	97	12.1	96	15.6	33	19.5				c
Asian	88	5.3	36	4.5	47	7.6	5	3.0				a
AI/AN	13	0.8	5	0.6	7	1.1	1	0.6				
Ethnicity									4.502	2	0.105	
Hispanic	109	6.5	62	7.3	42	6.5	5	2.9				
Non‐Hispanic	1567	93.5	793	92.77	606	93.5	168	97.1				
Sex									51.672	2	<0.001	
Male	541	31.7	219	25.1	232	35.4	90	50.8				a,b,c
Female	1165	68.3	654	74.9	424	64.6	87	49.2				a,b,c
Gender									53.329	6	<0.001	
Cis woman	1073	63.5	604	69.2	389	59.3	80	45.2				a,b,c
Cis man	494	29.2	202	23.1	206	31.4	86	48.6				a,b,c
Genderqueer	123	7.3	60	6.9	54	8.2	9	5.1				
Sexuality									25.679	6	<0.001	
Heterosexual	345	69.1	172	19.7	135	20.7	38	21.6				
Gay/lesbian	68	13.6	30	3.4	24	3.7	14	8.0				b
Bi/pansexual	51	10.2	40	4.6	10	1.5	1	0.6				
Queer (unspecified)	33	6.6	17	1.7	17	2.6	1	0.6				

^a^ Significance examined between age groups using Chi‐Square (*χ* ^2^) tests and Analysis of Variance (ANOVA).

^b^ Post‐hoc testing performed (Tukey and Bonferroni Pairwise Comparison of Proportions); a = adolescents significantly differed from emerging adults, b = emerging adults significantly differed from young adults, c = adolescents significantly differed from young adults.

Clinical characteristics of the sample are presented in Table 2. Overdose/ingestion (*n* = 1,133, 66.4%) was the most common suicide attempt method and the majority of patients expressed intent to die in their attempt (*n* = 1,468, 89%). Of the full sample, 753 patients (44.1%) had an interrupted or aborted suicide attempt and 953 (55.9%) did not. After arriving at the hospital, 711 (41.7%) only received care in the ED, 515 (30.2%) were admitted to a medical unit (including intensive care units), 812 (47.6%) were admitted to a psychiatric unit, and 332 (19.5%) received care from both medical and psychiatric units after the ED. Most patients experienced no injury (*n* = 944, 55.3%) or a low level of injury (*n* = 502, 29.4%) as a result of their attempt, and most experienced either no medical intervention (*n* = 1,101, 64.5%) or a moderate level of medical intervention (*n* = 345, 20.9%) during the encounter. On average, patients who were not transferred to an outside hospital for medical or psychiatric treatment had a 6.53‐day (SD = 7.2) LOS in the health system.

**TABLE 2:** Clinical characteristics by age group.

	Full sample (*n* = 1706)		Adolescents (*n* = 873, 51.2%)		Emerging adults (*n* = 656, 38.5%)		Young adults (*n* = 177, 104%)		Significance^a^			
	*n*	%	*n*	%	*n*	%	*n*	%	*F* or *χ* ^2^	*df*	*p*	Post hoc^b^
Past psychiatric history												
History of suicide attempt	970	56.9	468	53.6	397	60.5	105	59.3	7.78	2	0.020	a
1 prior attempt	197	12.1	105	12.4	71	11.4	21	12.8				
2–5 prior attempts	613	37.5	297	35.1	252	40.3	64	39.0				
6+ prior attempts	88	5.4	38	4.5	43	6.9	7	4.3				
History of self‐injury	1153	67.6	640	73.3	428	65.2	85	48	45.62	2	<0.001	a,b,c
Prior mental health diagnosis												
No prior diagnosis	132	7.7	73	8.4	49	7.5	10	5.6	1.62	2	0.444	
Depression	1281	75.1	660	75.6	494	75.3	127	71.8	1.19	2	0.551	
Anxiety	866	50.8	434	49.7	343	52.3	89	50.3	1.01	2	0.603	
ADHD	435	25.5	263	30.1	137	20.9	35	19.8	20.25	2	<0.001	a,c
Suicide attempt details												
Methods									73.49	16	<0.001	
Ingestion	1133	66.4	625	71.6	427	65.1	81	45.8				a,b,c
Hanging	113	6.6	66	7.6	31	4.7	16	9.0				
Firearm	20	1.2	7	0.8	9	1.4	4	2.3				
Jumping	54	3.2	25	2.9	23	3.5	6	3.4				
Cutting/stabbing	150	8.8	65	7.4	59	9.0	26	14.7				b
Monoxide	8	0.5	2	0.2	6	0.9	0	0				
Drowning	6	0.4	4	0.5	2	0.3	0	0				
Other	94	5.5	28	3.2	47	7.2	19	10.7				a,c
Multiple methods	128	7.5	51	5.8	52	7.9	25	14.1				b,c
Means already accessible	1527	89.5	815	93.4	559	85.2	153	86.4	28.42	2	<0.001	a,c
Self‐reported suicide intent	1468	89.0	768	90.4	549	86.7	151	91.0	5.59	2	0.061	
Attempt action												
Aborted attempt	421	24.7	186	21.3	193	29.4	42	23.7	13.37	2	0.001	a
Interrupted attempt	379	22.2	192	22.0	149	22.7	38	21.5	0.18	2	0.916	
Not stopped	953	55.9	515	59.0	339	51.7	99	55.9	8.13	2	0.017	a
Injuries/symptoms									5.15	6	0.524	
None	944	55.3	479	54.9	364	55.5	101	57.1				
Low	502	29.4	265	30.4	192	29.3	45	25.4				
Moderate	106	6.2	58	6.6	39	5.9	9	5.1				
Severe	154	9.0	71	8.1	61	9.3	22	12.4				
Suicide risk assessment												
C‐SSRS (M ± SD)	2.36	2.36	2.13	2.36	2.68	2.32	2.27	2.34	10.60	2	<0.001	a,b
Risk and protective factors												
Static risk	1092	64.0	536	61.4	422	64.3	134	75.7	13.13	2	0.001	b,c
Modifiable risk	1346	78.9	710	81.3	500	76.2	136	76.8	6.378	2	0.041	a
Imminent risk	772	45.3	386	44.2	287	43.8	99	55.9	9.13	2	0.010	b,c
Protective	1548	90.7	797	91.3	591	90.1	160	90.4	0.67	2	0.714	
Suicide risk level									17.64	4	0.001	
Low	53	3.6	22	2.9	26	4.8	5	3.3				
Moderate	437	30.1	202	26.6	193	35.5	42	28.0				a
Elevated/Heightened	963	66.3	536	70.5	324	59.7	103	68.7				a
Hospital encounter/treatment												
LOS in days (M, SD)	5.52	6.74	6.20	0.53	4.50	5.78	5.93	8.19	12.41	2	<0.001	a,b
Medical intervention required									14.10	6	0.029	
None	1101	64.5	554	63.5	430	65.5	117	66.1				
Mild	76	4.5	42	4.8	31	4.7	3	1.7				
Moderate	356	20.9	203	23.3	120	18.3	33	18.6				
Severe	173	10.1	74	8.5	75	11.4	24	13.6				
Hospital care												
Only ED	704	342	342	39.2	291	44.4	71	40.1	4.26	2	0.119	
Medical floor (e.g., ICU)	526	30.8	275	31.5	187	28.5	64	36.2	4.20	2	0.122	
Psychiatric unit	815	47.8	466	53.4	280	42.7	69	39.0	23.29	2	<0.001	a,c
Discharge disposition									21.80	10	0.016	
Home	961	59.1	510	60.4	354	57.4	97	58.8				
Home + PHP/IOP	202	12.4	120	14.2	67	10.9	15	9.1				
Residential treatment	44	2.7	24	2.8	12	1.9	8	4.8				
Another hospital (inpt.)	416	25.6	188	22.2	184	29.8	44	26.6				a

^a^ Significance examined between age groups using Chi‐Square (*χ* ^2^) tests and Analysis of Variance (ANOVA).

^b^ Post‐hoc testing performed (Tukey and Bonferroni Pairwise Comparison of Proportions); a = adolescents significantly differed from emerging adults, b = emerging adults significantly differed from young adults, c = adolescents significantly differed from young adults.

### Differences by age group

The average age of participants by age group was 15.59 (SD = 1.06) for Adol, 20.87 (SD = 2.25) for EA, and 27.56 (SD = 1.15) for YA. Significant differences by age group were found for several demographic characteristics and numerous clinical characteristics. Sex (*χ* ^2^(2) = 51.67, *p* < 0.001), gender (*χ* ^2^(6) = 53.33, *p* < 0.001), sexuality (*χ* ^2^(8) = 28.12, *p* < 0.001), and race (*χ* ^2^(6) = 19.07, *p* < 0.01) significantly differed by age group. Female sex was most prevalent among Adol (74.9%) and male sex was most prevalent among YA, in comparison to all other groups. Cisgender women (69.2%) were most often in the adolescent age group, cisgender men (48.6%) in the young adult group, and transgender or nonbinary gender non‐conforming (8.2%) in the emerging adult group, in comparison to all other groups. The adolescent group had the highest proportion of white patients (82.8%) and EA had the highest proportion of racial diversity among patients (24.3%) than other age groups.

As for clinical characteristics, significant differences by age group were found for suicide attempt method (*χ* ^2^(16) = 73.49, *p* < 0.0001), history of non‐suicidal self‐injurious behavior (*χ* ^2^(2) = 52.75, *p* < 0.001), interrupted/aborted attempt (*χ* ^2^(2) = 8.13, *p* < 0.05), history of suicide attempt (*χ* ^2^(2) = 7.45, *p* < 0.05), encounter locations (*χ* ^2^(4) = 17.23, *p* < 0.01), psychiatric unit admissions (*χ* ^2^(2) = 21.97, *p* < 0.001), length of hospital stay (*F*(2, 1694) = 12.41, *p* < 0.001), suicide assessment score (C‐SSRS) upon arrival (*F*(2, 1703) = 58.17, *p* < 0.001), discharge disposition (*χ* ^2^(8) = 22.53, *p* < 0.01), and provider‐determined risk level (*χ* ^2^(4) = 17.64, *p* < 0.01). Overdose/ingestion as an attempt method was most prevalent among Adol (71.6%), followed by EA (65.1%), and YA (45.8%). Hanging, firearms, cutting/sharp objects, and use of multiple methods were highest among YA (9%, 2.3%, 14.7%, and 14.1%, respectively), while jumping was most prevalent among EA, all in comparison to all other groups. A history of non‐suicidal self‐injury was most prevalent among Adol (69.1%), followed by EA (57.6%) and YA (52.4%). EA had the highest proportion of interrupted/aborted attempts (48.3%) than other age groups and Adol had the highest proportion of not interrupted/aborted attempts (59%) than other age groups.

The majority of patients in each group had a prior suicide attempt to their hospital encounter and among those with a history of attempt, YA had the largest prevalence (68.4%). Importantly and unsurprisingly, this greater prevalence is impacted by a longer duration of life in comparison to other age groups. The receipt of emergency care without admission was experienced most often by EA (44.5%) and the largest proportion of patients who were admitted on a psychiatric unit within their encounter were Adol (53%), followed by EA (42.7%), and YA (39%). The longest hospital stay was an average of 6.20 days (SD = 6.98) among Adol and the shortest was 4.50 days (SD = 5.78) among EA. Adol were most often discharged to the community (72.7%), EA to an outside medical or psychiatric hospital (28.6%), and YA to a residential treatment center/facility (4.6%). Pertaining to suicide attempt risk factors, YA were found to have the highest prevalence of both static (*χ* ^2^(4) = 13.13, *p* < 0.001) and imminent (*χ* ^2^(2) = 9.12, *p* < 0.05) factors, while Adol were noted to have the highest prevalence of modifiable factors (*χ* ^2^(2) = 6.38, *p* < 0.05). The highest C‐SSRS total score endorsed during the hospital encounter was 2.68 (SD = 2.32) among EA and the lowest was 2.13 (SD = 2.36) among Adol. Although most patients across all age groups were determined to be at a level of elevated and high suicide risk, more EA were identified to have a moderate level of risk (35.5%) than any other age group.

### Post hoc findings

Post hoc testing of demographic characteristic findings indicated that there were significantly more White Adol than White EA, more Asian EA than Asian Adol, and more Black YA than Black Adol (*p* = 0.006). As for sex and gender, the prevalence of cisgender males significantly increased from Adol to EA (*p* < 0.001), and from EA to YA (*p* < 0.001), while cisgender females significantly decreased as groups grew in age (*p* < 0.001).

Pertaining to clinical characteristics, significantly more EA had a history of suicide attempt than Adol (*p* = 0.02, *V* = 0.07), and history of self‐injurous behavior significantly decreased as groups grew in age (*p* < 0.001, *V* = 0.16). Ingestion as a method significantly decreased as groups grew in age (*p* < 0.001, *V* = 0.15), YA used multiple methods more than other groups (*p* < 0.001, *V* = 0.15), Adol more often had access to their suicide means (*p* < 0.001, *V* = 0.13), and EA aborted less often than Adol (*p* = 0.001, *V* = 0.09). Adol also had a higher prevalence of not stopping (i.e., aborting or interrupting) during the attempt than EA (*p* = 0.001, *V* = 0.09).

As for the C‐SSRS, EA had significantly higher scores than Adol and YA (*p* < 0.001, *η* ^2^ = 01), Adol had more modifiable risk factors than EA (*p* = 0.04, *V* = 0.06), and suicide risk level was more often moderate for EA (than Adol; *p* = 0.001, *V* = 0.08) and elevated for Adol (than EA; *p* = 0.001, *V* = 0.08). Lastly, the LOS was significantly shorter for EA than Adol and YA (*p* < 0.001, *η* ^2^ = 0.01), Adol more often received care from a psychiatric unit than other groups (*p* < 0.001, *V* = 0.12), and EA were more often transferred to another hospital than other groups (*p* = 0.001, *V* = 0.082).

## DISCUSSION

Suicide is an urgent public health concern and leading cause of death for transitional aged youth and young adults. Given many clinical resources and research implications focus on either pediatric or adult populations, there are limited understandings of risk for suicide attempt during the transition from adolescence to adulthood. This study sought to identify demographic and clinical characteristics of individuals who attempted suicide within the developmental timeframes of three specific age groups: adolescence (Adol), EA, and YA. Significant differences were found in various demographic and clinical characteristics across the three age groupings. For demographic characteristics, more racial diversity was present among emerging and young adults, and the prevalence of male patients increased with age. For clinical characteristics, more emerging adults had a history of suicide attempt and self‐injurous behavior was found to decrease with age. Ingestion was less often a suicide method as age grew across groups, adolescents more often had access to suicide means, and adolescents most often did not stop during their attempt and emerging adults aborted less often. Adolescents and young adults had lower C‐SSRS scores than emerging adults, adolescents had more modifiable risk factors, and suicide risk level was more often determined to be moderate for emerging adults and elevated for adolescents. As for their hospital encounter, LOS was shorter for emerging adults, adolescents most often were admitted to a psychiatric unit, and emerging adults were most often transferred to another hospital. Results highlight the dynamic and complex nature of suicide risk during developmental transitions, especially in the more recently proposed EA period.

Study implications expand upon well‐established literature that distinguishes EA as a developmental period separate from adolescence and adulthood (Arnett, 2000), as well as highlighting the complexity of identifying and managing suicide risk during this stage of life. First, there was significantly more racial and gender diversity in EA than in either of the other groups, with significantly more cis women in Adol and significantly more cis men in YA. This may suggest that developmental changes that occur during EA may exacerbate the already heightened risk for suicide associated with some racial and gender identities (CDC, 2024a, 2024b). Additionally, despite having the highest C‐SSRS scores, EA were most often labeled as being in a moderate risk level. EA also had the shortest LOS out of the three groups, despite not having any significant difference in the medical severity of their attempts. Given those at moderate risk often require greater assessment to clarify and determine the most appropriate course of treatment (Fiedorowicz et al., 2010), the discrepancies between C‐SSRS (self‐report), suicide risk level (provider‐labeled), and LOS may exemplify need for more tailored assessment tools that can more accurately assess for risk in this population.

In addition to improving risk assessment within this population, tailoring interventions to support those who have been identified to be at heightened risk is also critical. While well‐established approaches to suicide prevention like gatekeeper training (Gould & Kramer, 2001; Mann et al., 2005) and lethal means restriction (Bryan et al., 2011; Stanley & Brown, 2012) may be effective in reducing suicide risk for this population, the unique aspects of this developmental stage may necessitate further tailoring and targeted implementation to effectively reach this population. Interventions that encourage individuals at risk for suicide to limit their access to lethal means have been shown effective in both adolescents (Brent et al., 2000; McManus et al., 1997) and young adults (McPhedran & Baker, 2012; Stanley et al., 2020). For example, research shows that parents of youth receiving psychiatric care in the ED who received lethal means counseling were more likely to reduce their child's access to lethal means than parents who did not receive the counseling (Brent et al., 2000; McManus et al., 1997). However, as living arrangements and social supports evolve during EA, some young adults may be more likely to gain access to lethal means and less likely to have that access restricted than they were when living with parents or guardians. Therefore, identifying effective strategies that ensure lethal means restriction can be accomplished across developmental stages is crucial to prevent suicide death in this population.

While the ever‐changing nature of EA may pose barriers to these well‐established prevention strategies, there are also many components of the developmental period that may actually facilitate these efforts. Specifically, EA is hallmarked by expanding social networks and increased peer‐to‐peer connection (Wrzus et al., 2013), and therefore programs like gatekeeper training, which equip nonprofessionals with the skills to recognize those at risk and connect them to appropriate care (Mann et al., 2005), may actually have an increased likelihood of preventing suicide behavior. Still, most investigations of GKT in this population have focused on traditional college populations (Lipson et al., 2014), and while higher education is a part of EA for some, many EA do not pursue higher education, so future studies should consider other avenues to train gatekeepers in those young peoples' lives. Given the unique barriers and opportunities presented by the dynamic developmental period, further research is needed to identify the best approaches to identify risk and intervene accordingly.

Many young people report abrupt transitions from pediatric to adult mental healthcare services (Jivanjee & Kruzich, 2011) which may lead to many EA not having a natural connection to be guided to professional care (e.g., risk being identified at school) and also many not having access to mental healthcare at a time of heightened need. Research evaluating the effectiveness of interventions that aid young people through this transitional period is minimal, likely due to the overall lack of interventions and programs attempting to bridge this gap (Paul et al., 2015). Further, when EA do seek help for suicide thoughts and behavior or other mental health concerns, it is important that tailored, relevant, and individualized care is provided. Prior research indicates that providing EA with outpatient mental health programs geared toward their developmental period increases their service utilization compared to EA engaged in general adult outpatient programming (Gilmer, 2012). This may suggest that tailoring treatment of EA in an inpatient setting may also have the potential to improve patients' experiences and outcomes when compared to generalized adult mental healthcare. Even when they receive care in the same environment as a broader population (i.e., all adults), creating spaces for EA to connect with peers similar in age and experience within the broader environment or program may have the potential to enhance the care they receive.

There are several limitations of this study to consider. First, data were secondary in nature and collected retrospectively, therefore there were constraints in assessment of constructs. e.g., while some variables of interest were measured and documented in alignment with a standardized scale in the EHR (e.g., C‐SSRS), many areas of documentation had data structures of a construct being present or not present (i.e., binary prevalence of suicide attempt mean proximity). This overall limits the depth and detail of information, in addition to a limitation of some variables not being evaluated with standardized scales. Additionally, variations were evident across provider documentation (e.g., some providers shared more or less detail in notes) and impacted the degree of details that were collected for all constructs. Second, and related to the EHR sample, only patients who survived a suicide attempt and presented to this particular hospital setting were included in this study, therefore the findings cannot be extrapolated to those who attempt suicide and don't seek emergency care, present at another medical facility after their attempt, or died by suicide prior to arriving to the hospital. Third, and also related to the sample, comparisons of age groups did not factor in the age of suicide ideation and behavior onset given secondary data constraints. Our future investigative plans that involve prospective research including longitudinal data collection to examinto clinical trajectories over time as it relates to suicide behavior. Fourth, the number of patients in the YA group were relatively small (*n* = 177) in comparison to the Adol and EA groups (*n* = 873 and *n* = 656, respectively), making unequal sized groupings. It is important to interpret the findings within the context of knowledge that unequally sized groupings can lead to unequal variances between samples and loss of statistical power. Lastly, the study was cross sectional in nature and examined data of patients' most recent suicide attempt ED visit. Future longitudinal research is needed to examine data regarding fluctuations in suicide risk, suicide thoughts and behavior, and service utilization over time.

In sum, this study identified a number of clinical and demographic differences between those who attempt suicide during EA compared to patients who attempted suicide in either adolescence or YA. In this unique and impactful transitional period, suicide risk in the EA population is impacted by numerous developmental, social, educational, and occupational factors, therefore finding ways to reduce risk among those who have made a suicide attempt is crucial. Taken together, findings highlight the complexity of this developmental period and offer support for clinical considerations for this population's unique needs for engagement, assessment, and intervention. As researchers and clinicians work toward findings ways to better care for youth and young adults at risk for suicide, further investigations into the differences in characteristics between adolescents, emerging adults, and young adults are needed to better understand suicide behavior, more effectively intervene for those at risk, and ultimately prevent suicide death. Future longitudinal research is needed to examine fluctuations in factors that may drive suicide thoughts and behavior during these developmental transitions and develop and evaluate tailored assessments and interventions to better identify and treat emerging adults with suicidal thoughts and behavior.

## AUTHOR CONTRIBUTIONS

**Nicholas M. Brdar:** Conceptualization; data curation; formal analysis; project administration; supervision; writing—original draft; writing—review and editing. **Natasha K. Matta:** Data curation; writing—original draft; writing—review and editing. **Chloe A. Miner:** Data curation; writing—original draft; writing—review and editing. **Mubashshir R. Bhuiyan:** Data curation; writing—original draft; writing—review and editing. **Irmgard G. Pallas:** Data curation; writing—original draft; writing—review and editing. **Anna L. Bickersteth:** Data curation; writing—original draft; writing—review and editing. **Hailey G. Prokop:** Data curation; writing—original draft; writing—review and editing; **Lindsay A. Bornheimer:** Conceptualization; data curation; formal analysis; investigation; methodology; resources; software; supervision; writing—original draft; writing—review and editing.

## CONFLICT OF INTEREST STATEMENT

The authors declare no conflicts of interest.

## ETHICAL CONSIDERATIONS

This study was approved by the University Michigan Medical School Institutional Review Board (IRB) and determined to be exempt from ongoing IRB review.

## Data Availability

The data used in this study are available from the corresponding author upon reasonable request.
